# Sodium Stibogluconate (SSG) & Paromomycin Combination Compared to SSG for Visceral Leishmaniasis in East Africa: A Randomised Controlled Trial

**DOI:** 10.1371/journal.pntd.0001674

**Published:** 2012-06-19

**Authors:** Ahmed Musa, Eltahir Khalil, Asrat Hailu, Joseph Olobo, Manica Balasegaram, Raymond Omollo, Tansy Edwards, Juma Rashid, Jane Mbui, Brima Musa, Abuzaid Abdalla Abuzaid, Osama Ahmed, Ahmed Fadlalla, Ahmed El-Hassan, Marius Mueller, Geoffrey Mucee, Simon Njoroge, Veronica Manduku, Geoffrey Mutuma, Lilian Apadet, Hudson Lodenyo, Dedan Mutea, George Kirigi, Sisay Yifru, Getahun Mengistu, Zewdu Hurissa, Workagegnehu Hailu, Teklu Weldegebreal, Hailemariam Tafes, Yalemtsehay Mekonnen, Eyasu Makonnen, Serah Ndegwa, Patrick Sagaki, Robert Kimutai, Josephine Kesusu, Rhoda Owiti, Sally Ellis, Monique Wasunna

**Affiliations:** 1 Institute of Endemic Diseases, University of Khartoum, Khartoum, Sudan; 2 Addis Ababa University, Addis Ababa, Ethiopia; 3 Makerere University, Kampala, Uganda; 4 Médecins Sans Frontières-Holland, Amsterdam, The Netherlands; 5 Drugs for Neglected Diseases initiative (DNDi), Geneva, Switzerland; 6 MRC Tropical Epidemiology Group, London School of Hygiene and Tropical Medicine, London, United Kingdom; 7 Centre for Clinical Research, Kenya Medical Research Institute, Nairobi, Kenya; 8 Faculty of Medicine, Gedaref University, Gedaref, Sudan; 9 Gondar University, Gondar, Ethiopia; 10 Arba Minch Hospital, Regional Health Bureau of SNNPR State, Arba Minch, Ethiopia; 11 University of Nairobi, Nairobi, Kenya; 12 Amudat Hospital, Amudat, Uganda; London School of Hygiene and Tropical Medicine, United Kingdom

## Abstract

**Background:**

Alternative treatments for visceral leishmaniasis (VL) are required in East Africa. Paromomycin sulphate (PM) has been shown to be efficacious for VL treatment in India.

**Methods:**

A multi-centre randomized-controlled trial (RCT) to compare efficacy and safety of PM (20 mg/kg/day for 21 days) and PM plus sodium stibogluconate (SSG) combination (PM, 15 mg/kg/day and SSG, 20 mg/kg/day for 17 days) with SSG (20 mg/kg/day for 30 days) for treatment of VL in East Africa. Patients aged 4–60 years with parasitologically confirmed VL were enrolled, excluding patients with contraindications. Primary and secondary efficacy outcomes were parasite clearance at 6-months follow-up and end of treatment, respectively. Safety was assessed mainly using adverse event (AE) data.

**Findings:**

The PM versus SSG comparison enrolled 205 patients per arm with primary efficacy data available for 198 and 200 patients respectively. The SSG & PM versus SSG comparison enrolled 381 and 386 patients per arm respectively, with primary efficacy data available for 359 patients per arm. In Intention-to-Treat complete-case analyses, the efficacy of PM was significantly lower than SSG (84.3% versus 94.1%, difference = 9.7%, 95% confidence interval, CI: 3.6 to 15.7%, p = 0.002). The efficacy of SSG & PM was comparable to SSG (91.4% versus 93.9%, difference = 2.5%, 95% CI: −1.3 to 6.3%, p = 0.198). End of treatment efficacy results were very similar. There were no apparent differences in the safety profile of the three treatment regimens.

**Conclusion:**

The 17 day SSG & PM combination treatment had a good safety profile and was similar in efficacy to the standard 30 day SSG treatment, suggesting suitability for VL treatment in East Africa.

**Clinical Trials Registration:**

www.clinicaltrials.gov NCT00255567

## Author Summary

Visceral leishmaniasis (VL) is a parasitic disease with about 500,000 new cases each year and is fatal if untreated. The current standard therapy involves long courses, has toxicity and there is evidence of increasing resistance. New and better treatment options are urgently needed. Recently, the antibiotic paromomycin (PM) was tested and registered in India to treat this disease, but the same dose of PM monotherapy evaluated and registered in India was not efficacious in Sudan. This article reports the results of a clinical trial to test the effectiveness of injectable PM either alone (in a higher dose) or in combination with sodium stibogluconate (SSG) against the standard SSG monotherapy treatment in four East African countries—Sudan, Kenya, Ethiopia and Uganda. The study showed that the combination of SSG &PM was as efficacious and safe as the standard SSG treatment, with the advantages of being cheaper and requiring only 17 days rather than 30 days of treatment. In March 2010, a WHO Expert Committee recommended the use of the SSG & PM combination as a first line treatment for VL in East Africa.

## Introduction

The parasitic disease visceral leishmaniasis (VL) has an incidence of 500,000 new cases annually occurring primarily in India, Bangladesh, Nepal, Sudan, and Brazil and is fatal if untreated [1]. However, it is also an important disease in several other East African countries, with an incidence rate of 30,000 cases per year and a mortality rate of 4,000 deaths per year [2], [3].

VL treatment options in East Africa are primarily limited to the antimonial sodium stibogluconate (SSG), which is efficacious, but requires 4 weeks of hospitalization for daily intra-muscular injections and has been associated with serious adverse events such as cardiotoxicity; a concern in areas of HIV co-infection [3], [4], [5]. In India, leishmania parasites have developed resistance to SSG, with up to 65% of the population in the hyper endemic region of Bihar being unresponsive [6], [7]. SSG unresponsiveness is emerging in East Africa and treatment with a combination of SSG & PM may limit the spread of the emerging resistant strains of leishmania parasites [8].

The efficacy of paromomycin sulphate (PM) monotherapy for the treatment of VL has been demonstrated in India, where it is now registered for the treatment of VL [9] and the safety and efficacy of the combination of SSG and PM has been demonstrated in trials in India and a small Kenyan study [10], [11]. A large case series of 4,263 VL patients carried out by Médecins sans Frontières – Holland (MSF) in South Sudan showed that treating patients with a combination of SSG & PM for 17 days yielded better results than treating them with SSG alone: the initial cure rate was 97% for SSG & PM for 17 days versus 92% for SSG alone for 30 days [12].

For registration of PM and evaluation of the SSG & PM combination therapy throughout East Africa, efficacy and safety data were required from a Phase III trial. A multi-centre phase III trial has been conducted in six clinical trials sites in 4 East Africa countries (Ethiopia, Kenya, Sudan and Uganda). The trial started in 2004 with three arms; SSG monotherapy (20 mg/kg/day for 30 days: reference arm), PM monotherapy (15 mg/kg/day for 21 days) and SSG & PM combination (SSG: 20 mg/kg/day, PM: 15 mg/kg/day both given for 17 days).

The aim was to compare safety and efficacy of PM monotherapy and SSG & PM combination therapy with standard SSG treatment. An interim analysis showed that the PM monotherapy had an efficacy of <50% parasite clearance 6 months after the end of treatment in Sudan [13]. This arm was discontinued while a separate dose-finding trial of alternative PM regimens was conducted in Sudan [14]. The original Phase III trial was then restarted with a higher PM monotherapy dose (20 mg/kg/day for 21 days), while the other two arms remained unchanged.

The objectives remained the same; to compare the efficacy and safety of PM monotherapy and SSG & PM combination therapy to SSG. The results of this trial are reported here.

## Methods

### Ethics statement

The trial was conducted in accordance with the Declaration of Helsinki (2002 version) relating to the conduct of research on human subjects and followed the International Committee on Harmonization guidelines for the conduct of clinical trials. All trial site personnel received training in Good Clinical Practice (GCP).

The relevant ethics committees from each country approved the study and the details are listed in the attached supporting text document. Patients and their legal guardians (if they were minors) provided signed informed consent prior to being randomized to the different treatment arms. GCP-trained monitors recruited from all four participating countries regularly monitored the trial at all sites.

### Design

An open label, parallel-arm multi-centre individually randomized controlled trial.

### Participants

Patients were enrolled from six clinical trials sites: Médecins Sans Frontières (MSF) Holland treatment centre, Um el Kher, Gedaref State, Sudan; Ministry of Health Hospital Kassab, Gedaref State, Sudan; Gondar University Hospital, Amhara State, Northern Ethiopia; Arba Minch Hospital, SNNPR state, Southern Ethiopia; Centre for Clinical Research, Kenya Medical Research Institute (KEMRI), Nairobi, Kenya; and Amudat Hospital, Nakapiripirit Region, Uganda.

**Figure 1:**
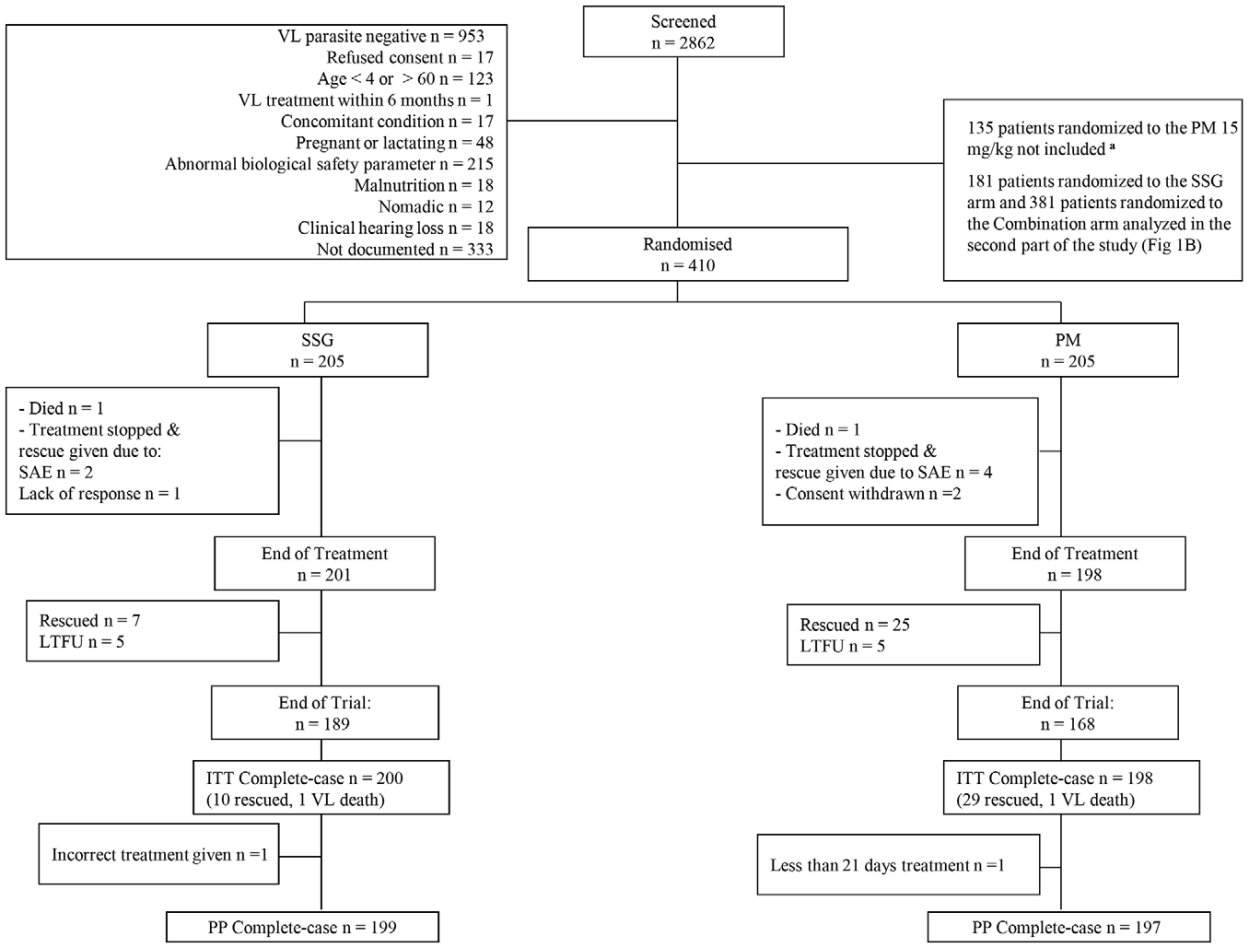
CONSORT Patient Flowchart – SSG *vs.* PM. SSG, sodium stibogluconate; PM, paromomycin sulphate; SAE, serious adverse event; LTFU, loss to follow-up; ITT, intention-to-treat; PP, per protocol. Patients included in the SSG (20 mg/kg/day for 30 days) vs. PM (20 mg/kg/day for 21 days) arms; **^a^** data from these patients were previously reported [14].

**Figure 2:**
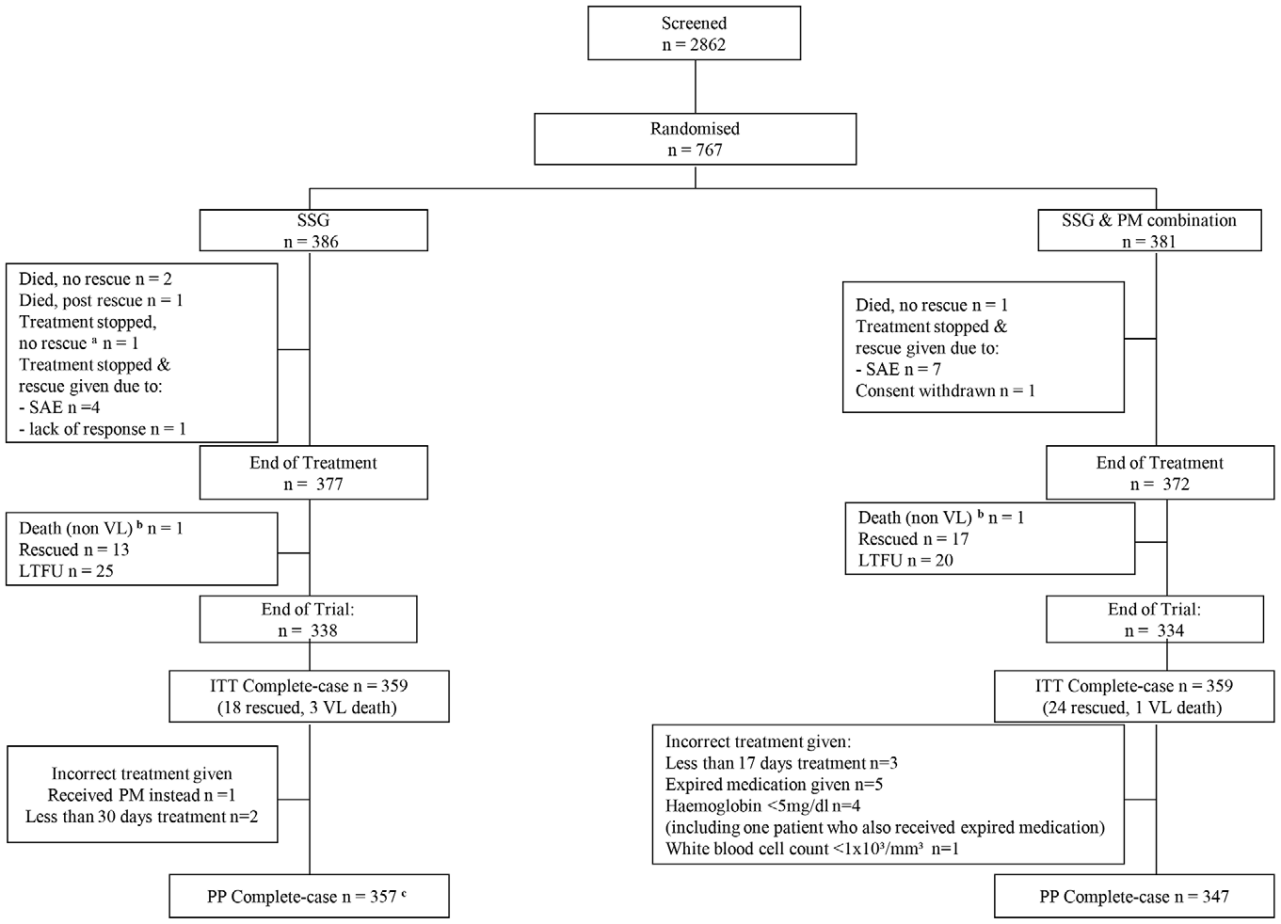
CONSORT Patient Flowchart – SSG *vs.* SSG&PM. SSG, sodium stibogluconate; PM, paromomycin sulphate; SAE, serious adverse event; LTFU, loss to follow-up; ITT, intention-to-treat; PP, per protocol. Patients included in the SSG (SSG at 20 mg/kg/day for 30 days) vs. SSG & PM combination (SSG at 20 mg/kg/day & PM at 15 mg/kg/day for 17 days) arms; **^a^** patient was diagnosed with tuberculosis and was removed from the study before the end of treatment; **^b^** patient died from non-VL causes; **^c^** patient with deviation also had a missing outcome value and was already excluded from the ITT analysis.

Inclusion and exclusion criteria have been described previously [13]. Briefly, patients aged 4–60 years with parasitologically confirmed VL were included, but patients with very severe VL or those with contraindications were excluded (Figures 1 and 2).

### Interventions

The three arms were SSG monotherapy (20 mg/kg/day for 30 days: reference arm), PM monotherapy (20 mg/kg/day for 21 days) and SSG & PM combination (SSG: 20 mg/kg/day, PM: 15 mg/kg/day both administered for 17 days).

Administration of PM (Gland Pharma, India) was intramuscular (IM). SSG (Albert David, India) was administered IM, or intravenous (IV) in Kenya. Patients requiring rescue medication were given liposomal amphotericin B, (manufactured as Ambisome®, Gilead, USA) according to national dosage guidelines of the participating countries. Patients were hospitalized for treatment and weekly monitoring of clinical and biological parameters. Follow-up visits were conducted 3 months and 6 months post end of treatment (Figures 1 and 2).

### Outcome Measures

The primary efficacy endpoint was definitive cure, defined as parasite clearance from splenic, bone marrow or lymph node tissue aspirates 6 months after the end of treatment. Any patient who died from VL, received rescue medication during the trial, or had parasites detected at the 6-month assessment was considered a treatment failure. The secondary efficacy endpoint was parasite clearance from tissue aspirates at the end of treatment (SSG: day 31, PM: day 22, SSG & PM: day18). Treatment failure at the end of treatment was defined as death or receipt of rescue medication during initial hospitalization or presence of parasites at end of treatment necessitating rescue treatment. The presence of parasites at the end of the treatment, subsequently cleared without need for rescue treatment was considered a treatment success for primary outcome (definitive cure at 6 months follow-up), but a treatment failure for secondary outcome (cure at end of treatment). Slow responders were defined as patients with detectable parasites at end of treatment and parasite clearance at 6 months follow-up, without need for rescue treatment at any time. Parasitology was performed and reported according to an approved World Health Organization (WHO) method [1]. The numbers of parasites in slide fields were counted under oil emersion at 100× magnification and counts recorded.

### Other Data Collection

Safety was evaluated based on the occurrence of adverse events (AE), laboratory parameters (haematology and biochemistry), electrocardiogram (ECG) readings, and audiometry. AEs were classified according to the Medical Dictionary for Regulatory Activities (MedDRA) version 10 [15]. A treatment emergent AE (TEAE) was defined as an AE with onset between the first day of treatment and 30 days after end of treatment.

ECGs were performed at all sites using a portable self-reporting ECG machine (Cardiofax, Model ECG 9620, Nihon Kohden) with patients resting supine on their beds. Trial physicians reviewed tracings and reported any abnormality.

Post-kala-azar dermal Leishmaniasis (PKDL) was recorded actively as an adverse event during patient follow-up or reported directly by the patients in between follow-up dates.

Audiometric testing was performed at all trial sites except Um el Kher using Voyager 522 Portable Diagnostic Audiometer (Madsen, Taastrup, Denmark). In recruitment period 1, investigators reported audiometric data as normal, clinically insignificant or clinically significant [13]. In period 2, hearing levels were recorded in detail for each ear at six frequencies. The following definitions were used to measure abnormalities; 1) *disabling hearing impairment* (DHI): an average hearing level, over frequencies 500, 1000, 2000, 4000 Hz, of ≥31 dB in both ears for those <15 years and ≥41 dB for those aged ≥15 years; 2) *audiometric shift:* a change in hearing level from baseline of ≥25 dB at ≥1 threshold frequency or ≥20 dB at ≥2 adjacent threshold frequencies.

All patients were offered counselling and HIV testing in accordance to national guidelines at screening.

### Sample Size Determination

The trial was designed to have 90% power (β = 0.1) to detect, at the 5% significance level (α = 0.05), an absolute difference in efficacy of 15% between PM and SSG and 10% between SSG & PM and SSG regimens [16]. An 85% efficacy was assumed in the reference arm and adjusting for 10% HIV co-infection and 10% loss to follow-up at 6 months post end of treatment, it was estimated that 404 and 195 patients per arm were required for the respective comparisons.

Being HIV-positive was not an exclusion criteria but the original protocol stated that there was to be a sufficient number of patients for a subgroup analysis excluding HIV patients (if deemed necessary).

### Randomization

As described at the end of the Introduction, recruitment and randomisation was carried out during two periods. In the first period, patients were randomised to SSG or SSG & PM combination arms, as part of a randomisation into three arms. Data from the third arm, a lower dosage regimen of PM found to be ineffective are not included here. In the second period, randomisation continued into one of three arms; SSG, SSG & PM arms as per period 1 and a PM monotherapy arm at a higher dosage regimen than previously (see Introduction and Interventions sections.)

In recruitment period 2 (using the higher 20 mg/kg dose of PM), randomization into 3 arms was continued until the desired sample size was reached for the PM versus SSG comparison. Randomization was then continued into one of two arms (SSG or SSG & PM) until reaching the sample size for the SSG versus SSG & PM comparison. Um el Kher site participated in period 1 only and Amudat site in period 2 only (during the two-arm randomization).

A computer-generated randomization list was produced with stratification by centre and block sizes of 15 until recruitment in the PM arm was completed, and block sizes of 10 thereafter. Allocation was concealed using opaque, sequentially numbered sealed envelopes. The randomization list and envelopes were prepared and stored securely at the LEAP Data Centre, based at the trial co-ordination centre in Nairobi.

Blinding of patients and investigators was not possible due to the different treatment durations and additional placebo injections were considered inappropriate.

### Statistical Methods

Data were double-entered and validated in Epi-Info. Bespoke query generation programs were developed using Stata software, version 11 [17]. All statistical analyses were performed using Stata. Baseline data were summarized using mean and standard deviation (SD) or proportions where appropriate. Nutritional status was classified as normal, underweight, or severely underweight according to WHO Child Growth Standards in those <19 years and body mass index (BMI) in those ≥20 years [18].

### Analysis Populations

For the SSG *vs.* PM comparison, patient data from randomisation during period 2 are included in this comparison. For the SSG *vs* SSG & PM comparison, patient data from randomisation into these arms in periods 1 and 2 are included in this comparison.

Efficacy data were analysed according to Intention-to-Treat (ITT) and Per-Protocol (PP). The PP population excluded those with pre-specified major protocol deviations (i.e. consent withdrawal after taking a dose of study medication, receipt of under 70% or over 130% of the expected treatment dosage, or receipt of alternative treatment to that of random allocation). Missing efficacy data were handled in two ways for each analysis population; complete-case analysis, where patients with missing data were excluded and worst-case analysis, where missing outcomes were considered treatment failures.

Efficacy is measured as the percentage of patients cured per arm. The treatment effect is the difference in efficacy between each test treatment (PM or SSG & PM) and the reference (SSG). Unadjusted treatment effects were calculated with exact binomial 95% confidence intervals (CI). Adjusted treatment effects were obtained using generalized linear models with a binomial distribution and identity link function. To assess possible effects of centre, age group (<18 years and ≥18 years) and recruitment period on efficacy after accounting for treatment allocation, regression models including treatment but with and without the covariate of interest were compared using the likelihood ratio test (LRT).

Treatment emergent adverse event (TEAE) rates were calculated as the number of TEAE, divided by the person-days at risk for each arm, and comparisons made using rate ratios. The treatment emergent period was defined as between day 1 of treatment and 30 days after the pre-defined treatment period, inclusive, therefore person-time at risk was as follows; SSG arm: 60 days, PM: 51 days, SSG & PM: 47 days. An adverse drug reaction was defined where an investigator recorded a probable, possible or unlikely relationship between the AE and study drug for VL.

## Results

### Patient Population

The study was initiated in November 2004 and was completed in January 2010. A total of 2862 patients were screened for entry into the trial. Of these, 1755 were excluded (Figures 1 and 2), mainly due to negative parasitology. For the PM monotherapy versus SSG comparison, 205 patients per arm were recruited during period 2 (Figure 1). The total sample size for the SSG versus SSG & PM comparison was 386 patients in the SSG arm and 381 for SSG & PM (Figure 2): 135 patients per arm from period 1; 251 and 246 per arm respectively, from period 2.

Treatment arms were balanced for both comparisons with respect to demographic characteristics, vital signs, and physical measurements (combined arm data shown in Table 1). There were more male than female patients and more than 65% of patients were under the age of 18 years. All biological data except for nutritional status were balanced between arms at baseline; more patients in the PM and SSG & PM arms were classified as severely underweight but, overall combined percentages of underweight and severely underweight were balanced by arm. Overall, for all recruited patients, the HIV co-infection frequency was 1.4% (95% CI: 0.8–2.4%).

**Table 1:** Baseline Data.

		SSG	PM	SSG & PM
		N = 386	N = 205	N = 381
Centre	Ethiopia: Gondar, n (%)	60 (15.5)	15 (7.3)	60 (15.8)
	Ethiopia: Arba Minch, n (%)	45 (11.7)	15 (7.3)	45 (11.8)
	Kenya: KEMRI, n (%)	71 (18.4)	35 (17.1)	70 (18.4)
	Sudan: Um el Kher, n (%)	30 (7.8)	-	30 (7.9)
	Sudan: Kassab, n (%)	167 (43.3)	140 (68.3)	165 (43.2)
	Uganda: Amudat, n (%)	13 (3.4)	-	11 (2.9)
Age	Mean (SD)	15.3 (9.3)	15.3 (9.9)	16.1 (9.4)
	Age 4–17 years^a^, n (%)	259 (67.1)	143 (69.8)	246 (64.6)
	Age ≥18 years^a^, n (%)	127 (32.9)	62 (30.2)	135 (35.4)
Sex	Female, n (%)	105 (27.2)	80 (39.4)	108 (28.4)
	Male, n (%)	281 (72.8)	125 (61.0)	273 (71.6)
Anthropometry^b^	Weight (Kg)	33.5 (14.5)	33.1 (15.5)	34.2 (14.7)
	Height (m)	1.4 (0.2)	1.4 (0.2)	1.5 (0.2)
Vital Signs^b^	Body temperature (°C)	38.1 (1.1)	38.4 (1.0)	38.2 (1.1)
	Heart Rate (beats/min)	108.2 (16.1)	111.7 (14.4)	107.3 (15.9)
	Systolic BP (mm Hg)	96.3 (11.2)	96.1 (10.4)	97.3 (11.1)
	Diastolic BP (mm Hg)	61.5 (8.4)	59.8 (7.9)	61.6 (8.1)
Organ Size^b^	Spleen Size (cm)	8.1 (5.0)	7.7 (5.0)	8.0 (4.8)
	Liver Size (cm)	3.0 (2.6)	2.8 (2.7)	3.0 (2.6)
Nutritional Status^c^	Severely underweight, n (%)	61 (15.8)	60 (29.3)	105 (27.5)
	Underweight, n (%)	167 (43.3)	62 (30.2)	140 (36.8)
	Normal, n (%)	156 (40.4)	78 (38.6)	134 (35.2)
	Obese/overweight, n (%)	2 (0.5)	4 (2.0)	2 (0.5)
HIV Status^d^	HIV-positive, n (%)	5 (1.3)	0 (0)	9 (2.4)

SSG = sodium stibogluconate (20 mg/kg/day for 30 days); PM = paromomycin sulphate (20 mg/kg/day for 21 days); SSG & PM Combination treatment (SSG at 20 mg/kg/day plus PM at 15 mg/kg/day for 17 days);

a Patients 4–17 years old were classified as children and patients 18–60 years old were classified as adults.

b These are presented as mean (SD).

c Classification based on World Health Organization child growth standards in patients ≤19 years or using body mass index in those ≥20 years.

d 340 out of 386, 203 out of 205, and 335 out of 381 patients were tested for HIV in the SSG, PM and SSG & PM arms respectively.

### Compliance

In the population analysed for the SSG versus PM comparison (n = 205 per arm), one patient in each arm did not receive the correct treatment allocation (Figure 1). Two patients in the PM arm withdrew consent after 4 and 6 days of treatment. For the SSG versus SSG & PM analysis population, three (0.8%) patients in the SSG arm and eight (2.0%) in the SSG & PM arm received a partial or incorrect dose (Figure 2). One SSG & PM patient withdrew consent after 6 days on treatment. Patients who had their 6-month follow-up at or before 4.5 months after the end of treatment were considered lost to follow-up since these visits were too early to assess definitive cure. For the SSG versus PM comparisons, outcome data for one (0.5%) patient in the SSG arm and two (1.0%) in the PM arm were considered missing. For the SSG versus SSG & PM comparison, outcome data for 13 (6.5%) SSG patients and nine (2.5% )SSG & PM patients were treated as missing data based on early follow-up. Data for patients whose primary endpoint assessment was later than 6 months were included in the analysis.

### Efficacy: PM versus SSG

Efficacy in the SSG (reference) arm was 94% at 6 months after the end of treatment and 84% in the PM arm, according to the ITT complete-case population. All pre-specified primary endpoint analyses (ITT complete-case and worst-case, PP complete-case and worst-case) suggest that the efficacy of PM monotherapy was significantly lower than SSG - up to 17% less efficacious (Table 2). There were negligible differences in estimates of treatment effect and corresponding 95% CIs in these four pre-specified analyses. After adjustment for arm, efficacy did not differ between adults (≥18 years) and children (p>0.4 for both ITT and PP complete-case analyses).

**Table 2:** Paromomycin (PM) monotherapy versus Sodium Stibogluconate (SSG): Efficacy Data.

Number of patients analyzed^a^	Number (%) cured	Treatment effect^b^ (95% CI), p-value^c^
Six months follow-up:		
ITT: Complete Case Analysis^d^		
SSG: N = 200	188 (94.0)	9.7 (3.6–15.7), p = 0.002
PM: N = 198	167 (84.3)	
PP: Complete Case Analysis^d^		
SSG: N = 199	188 (94.5)	10.2 (4.2–16.2), p = 0.001
PM: N = 197	166 (84.3)	
ITT: Worst Case Analysis^e^		
SSG: N = 205	188 (91.7)	10.2 (3.7–16.8), p = 0.002
PM: N = 205	167 (81.5)	
PP: Worst Case Analysis^e^		
SSG: N = 204	188 (92.2)	10.8 (4.3–17.3), p = 0.001
PM: N = 204	166 (81.4)	
End of Treatment:		
ITT: Complete Case Analysis^d^		
SSG: N = 205	197 (96.1)	9.9 (4.4–15.3), p<0.001
PM: N = 203	175 (86.2)	

CI = confidence interval, ITT = Intention-to-Treat, PP = Per-Protocol.

a 205 patients were originally recruited to the PM arm, 386 to the SSG arm.

b Treatment effect: difference in efficacy between SSG and PM, percent scale with exact binomial 95% CI. Adjustment for centre was not possible due to only one failure in one centre.

c p-value from likelihood ratio test comparing binomial regression models with and without treatment.

d Complete-case analysis: patients with missing outcome data excluded from analysis.

e Worst-case analysis: missing outcomes assumed to be treatment failures.

There were 8 (4.0%) slow responders of the 198 ITT complete-case PM patient population at 6 months after the end of treatment and none in the SSG arm. Secondary endpoint treatment effects measured at the end of treatment were again very similar to 6 months primary endpoint data (Table 2).

### Efficacy: SSG & PM versus SSG

In ITT complete-case primary endpoint analyses, the efficacy of SSG was 94% and for SSG & PM, 91% (Table 3). No difference in efficacy was noted between treatments. After adjusting for arm, no additional differences in efficacy were found between centres, age groups or recruitment periods (p>0.1, Table 3).

**Table 3:** Sodium Stibogluconate (SSG) & Paromomycin (PM) versus SSG: Efficacy Data.

Number of patients analyzeda	Number (%)cured	Treatment effect^b^ (95% CI), p-value^c^	Centre p-value^d^	Age p-value^d^	Period p-value^d^
Six months follow-up:					
ITT: Complete Case Analysis^e^					
SSG: N = 359	337 (93.9)	2.5 (−1.3–6.3)	0.337	0.122	0.112
SSG & PM: N = 359	328 (91.4)	p = 0.198			
PP: Complete Case Analysis^e^		,			
SSG: N = 357	336 (94.1)	2.8 (−1.1–6.6)	0.286	0.080	0.064
SSG & PM: N = 347	317 (91.4)	p = 0.157			
ITT: Worst Case Analysis^f^					
SSG: N = 386	337 (87.3)	1.2 (−3.6–6.0)	<0.001	0.008	<0.001
SSG & PM: N = 381	328 (86.1)	p = 0.620			
PP: Worst Case Analysis^f^					
SSG: N = 383	336 (87.7)	1.8 (−3.0–6.7)	<0.001	0.007	<0.001
SSG & PM: N = 369	317 (85.9)	p = 0.460			
End of Treatment:					
ITT: Complete Case Analysis^e^			-	-	-
SSG: N = 385	366 (95.1)	1.9 (−1.4–5.3)			
SSG & PM: N = 378	352 (93.1)	p = 0.254			

CI = confidence interval, ITT = Intention-to-Treat, PP = Per-Protocol.

a 381patients were originally recruited to the SSG&PM arm, 386 to the SSG arm.

b Treatment effect: difference in efficacy between SSG and SSG & PM combination treatment, percent scale with exact binomial 95% CI.

c p-value from likelihood ratio test comparing binomial regression models with and without treatment.

d p-value from likelihood ratio test comparing binomial regression models with and without factor of interest, after adjustment for treatment allocation.

e Complete-case analysis: patients with missing outcome data excluded from analysis.

f Worst-case analysis: missing outcomes assumed to be treatment failures.

Worst-case analyses in the ITT and PP populations did suggest some additional variation by centre, age group and period after accounting for arm; due to some imbalance in losses to follow-up by age group and centre. However, treatment effects and corresponding 95% CIs were very similar in all four pre-specified primary endpoint analyses (Table 3).

In the SSG arm, 3 (0.8%) of 359 ITT complete-case analysis patients were slow responders, compared to 7 (1.9%) of the 359 SSG & PM patients. End of treatment secondary endpoint efficacy data were in agreement with primary endpoint data (Table 3).

### Safety

The proportion of patients with SAE and non-serious TEAEs was similar in comparisons of both test treatment regimens to SSG (Table 4). Approximately 3% of patients in each arm in each comparison experienced an SAE deemed to be an adverse drug reaction (Table 4). One death occurred during the treatment period in each arm in the SSG versus PM comparison. In the SSG & PM versus SSG comparison, there were 3 deaths during initial hospitalization and a death of unknown cause during follow-up in the SSG arm. In the SSG & PM arm, there was a treatment period death and an unrelated death during follow-up (Tables 4 and 5). Of the 5 cases of renal impairment, 3 led to death, whilst 2 resolved after some time. Patients were withdrawn from treatment in all cases. Important cardiac events occurred in two patients: one in the SSG-PM arm and one in the SSG arm. In the former, a long QT interval appeared on Day 7, leading to treatment withdrawal. The long QT interval resolved 3 days later. In the second case, the patient died due to cardiotoxicity on Day 11 of treatment.

**Table 4:** Serious and non-serious adverse events occurring during the study.

	SSG	PM^a^	SSG & PM^a^
	N = 386	N = 205	N = 381
N (%) of patients with at least one AE			
At any time	271 (70.2)	126 (61.5)	251 (65.9)
TEAEs^b^	237 (61.4)	107 (52.2)	207 (54.3)
N (%) of patients with an SAE^c^			
Total	17 (4.4)	8 (3.9)	16 (4.2)
TEAEs^b^	14 (3.6)	7 (3.4)	16 (4.2)
Adverse drug reactions^d^	10 (2.6)	6 (2.9)	13 (3.4)
Deathse	4 (1.0)	1 (0.5)	2 (0.5)
Total number of all TEAEs recorded	445	192	348
Total person-days at risk^f^	23160	10363	17866
TEAE Rate	0.019	0.019	0.019

SSG = sodium stibogluconate; PM = paromomycin sulphate; SSG & PM = combination treatment;

AE, adverse event; SAE, serious adverse event; TEAE, treatment emergent adverse event;

a There were two consent withdrawals in the PM arm (after 4 and 6 days on treatment) and 1 withdrawal in the SSG & PM arm (after 6 days on treatment) - data were therefore collected only up to the day of withdrawal for these patients.

b Treatment emergent adverse event is defined as onset being between day 1 of treatment and 30 days post end of treatment, inclusive.

c No patient experienced more than one SAE.

d Adverse drug reaction is defined as any adverse event the investigator recorded as having a probable, possible or unlikely relationship to the study drug.

e Cause of deaths were as follows: SSG: unknown (1), Acute Renal Failure (2), cardiotoxicity (1); PM: VL; SSG & PM: Pericarditis tuberculosis (1), malaria (1).

f Person-days at risk is defined as the treatment period per study drug regimen plus an additional 30 days post end of treatment.

**Table 5:** All Serious Adverse Events (non-related events and related adverse drug reactions) by System Organ Class (bold) and Preferred Term according to MedDRA.

System organ class and preferred MedDRA term	SSG		PM^b^		SSG & PM^d^	
	N = 386		N = 205		N = 381	
	*NR*	*SADR*	*NR*	*SADR*	*NR*	*SADR*
**Cardiac disorders**	**0**	**1**	**0**	**0**	**0**	**0**
Cardiotoxicity	0	1	0	0	0	0
**Gastrointestinal disorders**	**1**	**1**	**0**	**0**	**0**	**1**
Pancreatitis acute	0	0	0	0	0	1
Pancreatitis	0	1	0	0	0	0
Peritoneal haemorrhage	1	0	0	0	0	0
**General disorders and administrative site conditions**	**1**	**0**	**0**	**0**	**0**	**0**
Death^a^	1	0	0	0	0	0
**Hepatobiliary disorders**	**0**	**0**	**0**	**0**	**0**	**1**
Hepatic function abnormal^b^	0	0	0	0	0	1
**Immune system disorders**	**2**	**0**	**0**	**0**	**0**	**0**
Anaphylactic shock	2	0	0	0	0	0
**Infections and Infestations**	**1**	**2**	**2**	**2**	**3**	**0**
Abdominal sepsis^c^	0	1	0	0	0	0
Malaria^c^	0	1	0	0	1	0
Hepatitis A	0	0	0	1	0	0
Herpes Zoster	1	0	0	0	0	0
Otitis Media	0	0	1	0	0	0
Pericarditis tuberculosis	0	0	0	0	1	0
Pneumonia	0	0	0	0	1	0
Pulmonary tuberculosis	0	0	1	0	0	0
Visceral leishmaniasis	0	0	0	1	0	0
**Investigations**	**0**	**4**	**0**	**2**	**0**	**9**
Alanine amino transferase increased (ALT only)	0	0	0	0	0	1
Blood alkaline phosphatase increased (ALP only)	0	0	0	0	0	1
Blood amylase increased	0	1	0	0	0	0
Electrocardiogram QT prolonged	0	0	0	0	0	1
Hepatic enzymes increased (bilirubin, ALT/AST/ALP)	0	1	0	1	0	3
Transaminases increased (ALT/AST)	0	2	0	1	0	3
**Nervous system disorders**	**0**	**0**	**0**	**1**	**0**	**0**
Febrile convulsion	0	0	0	1	0	0
**Renal and urinary disorders**	**0**	**2**	**0**	**1**	**0**	**2**
Renal impairment	0	2	0	1	0	2
**Reproductive system and breast disorders**	**1**	**0**	**0**	**0**	**0**	**0**
Priapism	1	0	0	0	0	0
**Respiratory, thoracic and mediastinal disorders**	**1**	**0**	**0**	**0**	**0**	**0**
Epistaxis	1	0	0	0	0	0

MedDRA, Medical Dictionary of Regulatory Activities; SSG, sodium stibogluconate (20 mg/kg/day for 30 days); PM paromomycin sulphate (20 mg/kg/day for 21 days); SSG & PM (SSG 20 mg/kg/day & PM at 15 mg/kg/day for 17 days); NR, non-related Serious Adverse Events; SADR, Serious Adverse Drug Reaction.

a Death due to an unknown cause.

b Raised bilirubin/jaundice.

c Abdominal sepsis and malaria were considered as unlikely related to the drug by the investigators.

d 2 PM patients withdrew consent after 4 and 6 days on treatment and 1 SSG & PM patient after 6 days on treatment, no SAE reported prior to withdrawal.

Rates and rate ratios, adjusted for centre, in both comparisons show no difference in safety based on analysis of TEAEs; adjusted rate ratio between the SSG and PM arm: 1.13, (95% CI: 0.93 to 1.38, p = 0.225) and between the SSG and Combination arms: 1.01, (95% CI: 0.88 to 1.17, p = 0.993). All of the non-fatal SAEs in the SSG and Combination arms resolved by the 6-month follow-up and all except one (pulmonary tuberculosis) in the PM arm resolved by the 6-month follow-up.

Treatment emergent adverse drug reactions (TEADRs) occurring in ≥10% of patients in the PM arm were injection site pain (13.2%), increase in aspartate aminotransferase (10.7%), and epistaxis (13.2%). In the subset of SSG patients analysed in the SSG versus PM comparison, TEADRs occurring in ≥10% of patients were aspartate aminotransferase increases (10.2%) and epistaxis (11.2%). For the population in the SSG versus Combination arms, no TEADR occurred in ≥10% of patients in the larger group of SSG patients. In the Combination arm, the most common TEADRs were injection site pain (17.3%) and increases in aspartate aminotransferase (10.5%).

Two patients in the Combination arm and one in the SSG arm had abnormal ECG findings that were considered clinically significant at end of treatment. These were, respectively, QT-wave inversion in V1–V4, arrhythmia and QT interval prolongation, which had normalized by 6 months follow-up.

In the SSG vs. PM comparison, 26 (12.7%) out of 205 patients developed PKDL in the SSG arm and 18 (8.9%) out of 203 patients randomised to PM. In the SSG *vs* SSG & PM comparison, 48 (12.4%) out of 386 patients in the SSG group and 23 (6.1%) out of 380 patients in the SSG-PM group developed PKDL. Two patients were given SSG for PKDL during their three months follow-up visit. DHI was reported in one patient in the PM and one patient in the Combination arm at the end of treatment, both of which resolved by the 6-month follow-up. None of the patients in the SSG arm had DHI. Thirty-six patients had audiometric shift at end of treatment (11 patients in the SSG arm, nine in the PM arm, and 16 in the SSG & PM arm). Audiometric shifts had still not resolved at the 6-month follow-up in three of the SSG, four of the PM and eight of the Combination patients.

## Discussion

This phase III GCP-compliant RCT investigated the safety and efficacy of PM both as monotherapy (20 mg/kg/day for 21 days) and as short course treatment in combination with SSG (PM at 15 mg/kg/day and SSG at 20 mg/kg/day for 17 days) for VL treatment in four East African countries, with the ultimate goal of determining if the SSG & PM combination treatment has acceptable safety and efficacy profiles to support its introduction in the region.

### Study Limitations

Definitive cure at six months follow-up in patients treated with SSG or SSG & PM was comparable with greater than 90% efficacy, despite PM monotherapy having significantly lower efficacy (84% cured) compared to SSG. Efficacy of the 20 mg/kg/day PM monotherapy at the 33% higher dose used in this study was better than that of the 15 mg/kg/day dose used earlier [13] (6-month cure rate of 84% vs. 64%), and is consistent with the dose-finding study conducted by the authors in Sudan [14]. However, the efficacy at this higher dose was still lower than that of SSG alone. By contrast, studies performed in India had shown that the efficacy of PM was consistently >90% at 15 and 20 mg/kg day for 21 days [19], [20], with PM showing better efficacy than SSG (20 mg/kg/day for 30 days) in the Jha *et al.* study [19] and non-inferior efficacy compared with amphotericin (1 mg/kg/day every 2 days for 30 days) in the Sundar *et al.* study [20]. Pharmacological differences in the East African and Indian populations that may explain these results were explored and will be reported separately. Geographical variation in efficacy of PM seen for the lower daily dose (15 mg/kg) was not apparent in this study with the higher daily dosage (20 mg/kg), though it must be noted that sufficient numbers of patients were not enrolled at all sites to perform a by-site analysis.

Secondary endpoints were performed at different times for each of the treatments (day 18 for the combination, day 22 for PM and day 31 for SSG), assumed comparable by design but potentially leading to bias in clinical and parasitological evaluations. Similarly, lack of blinding also may have led to bias in reporting, especially once lack of PM efficacy at the 15 mg/kg dose was suspected. As numerous sites and countries were involved, differentiation of reporting, particularly of adverse events was possible. Nonetheless, using a standard primary endpoint at 6 months and an objective measurement of efficacy based on parasitology, high rates of follow up were achieved. This is reflected in the relatively robust and comparable findings of the ITT, per protocol, complete case and worst case analyses.

The trial was powered to evaluate efficacy at the primary endpoint of 6 months follow-up and had limited power to detect differences in safety outcomes. However, almost identical rates of TEAEs and proportions of patients with adverse drug reactions were observed in patients treated with each regimen in the trial. The study was not powered to perform a subgroup analysis in HIV-positive patients assuming a 10% co-infection rate and HIV positive patients were not excluded. HIV co-infection was lower than expected, which may be due to the relatively small number of patients enrolled in Northern Ethiopia, where up to 35% co-infection had previously been reported [21]. In this study, 3 out of 5 and 5 out of 9 HIV co-infected patients had parasite clearance at 6 months after treatment with SSG and SSG & PM respectively. It was not possible to conclude on the difference in toxicity of either treatment among HIV co-infected patients.

Almost all of the SAEs that emerged in the three arms during treatment had resolved by the 6-month follow-up. There was no evidence of any new or important safety events, in either the PM or Combination arm. Although slightly more audiometric shifts remained at the 6-month follow in the PM and SSG & PM arms compared with the SSG arm, the trial was not powered to test for differences. With a larger sample size, percentages of patients with shifts remaining may have been balanced. Although not statistically significant, three deaths in the SSG arm were considered to be treatment-related (cardiotoxicity and renal disorders), whereas there were no treatment-related deaths in the Combination arm.

### Conclusion

These results, together with those of a retrospective comparison of a 17 day regimen of SSG & PM versus 30 days of SSG alone carried out among 4,263 primary VL patients in South Sudan [12] support the use of a shorter course Combination therapy for VL in East Africa, which would be consistent with the long-term goal of reducing reliance on SSG monotherapy.

The reduced duration of treatment with the Combination compared with SSG (17 versus 30 days) will also reduce burden on hospitals and patients and other associated costs. The cost of drugs alone compares favourably for the Combination in comparison to SSG (44US$ versus 55.8 US$ respectively for a patient weighing 35 kg) [1]. Finally, the potential risk of development of parasite resistance to the treatment could be reduced.

In conclusion, our results show that SSG & PM combination treatment has comparable efficacy and safety profiles to conventional SSG monotherapy in a Phase III setting, and support its introduction for treatment of primary VL in East Africa.

## Supporting Information

Checklist S1 (DOC)

Protocol S1 (PDF)
