# Supplementary material for: Sodium Stibogluconate (SSG) & Paromomycin Combination Compared to SSG for Visceral Leishmaniasis in East Africa: A Randomised Controlled Trial
Source: PLoS Negl Trop Dis. 2012 Jun 19;6(6):e1674. doi: 10.1371/journal.pntd.0001674 (PMC3378617; doi:10.1371/journal.pntd.0001674)
Supplement: Checklist S1 — (DOC) [file pntd.0001674.s001.doc]

# PloS-M DOUGADOS

CONSORT **Checklist of items to include when reporting a randomized trial**
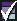


| PAPER SECTION And topic | Item | Description | Reported in |
| --- | --- | --- | --- |
| *METHODS*  *Randomization: Sequence generation and Allocation concealment* | 1 | [How participants were allocated to interventions](http://www.consort-statement.org/examples1.htm) (*e.g*., "random allocation", "randomized", or "randomly assigned"). | *METHODS*  *Randomization: Para1/2* |
| *INTRODUCTION* Background | 2 | [Scientific background and explanation of rationale.](http://www.consort-statement.org/examples2.htm) | *INTRODUCTION* |
| *METHODS* Participants | 3 | [Eligibility criteria for participants](http://www.consort-statement.org/examples3a.htm) and the [settings and locations where the data were collected](http://www.consort-statement.org/examples3b.htm). | *METHODS* Participants |
| METHODS  Interventions | 4 | [Precise details of the interventions intended for each group and how and when they were actually administered.](http://www.consort-statement.org/examples4.htm) | METHODS  Interventions |
| METHODS  Objectives | 5 | [Specific objectives and hypotheses](http://www.consort-statement.org/examples5.htm). | METHODS  Introduction (last para) |
| METHODS  Outcomes | 6 | [Clearly defined primary and secondary outcome measures](http://www.consort-statement.org/examples6a.htm) and, when applicable, any [methods used to enhance the quality of measurements](http://www.consort-statement.org/examples6b.htm) (*e.g.*, multiple observations, training of assessors). | METHODS  Outcomes |
| METHODS  Sample size | 7 | [How sample size was determined](http://www.consort-statement.org/examples7a.htm) and, when applicable, [explanation of any interim analyses and stopping rules](http://www.consort-statement.org/examples7b.htm). | METHODS  Sample size determination |
| METHODS  Randomization -- Sequence generation | 8 | [Method used to generate the random allocation sequence, including details of any restrictions](http://www.consort-statement.org/examples8a.htm) (*e.g*., blocking, stratification) | METHODS  Randomization |
| METHODS  Randomization -- Allocation concealment | 9 | [Method used to implement the random allocation sequence](http://www.consort-statement.org/examples9.htm) (*e.g*., numbered containers or central telephone), clarifying whether the sequence was concealed until interventions were assigned. | METHODS  Randomization |
| METHODS  Randomization -- Implementation | 10 | [Who generated the allocation sequence, who enrolled participants, and who assigned participants to their groups.](http://www.consort-statement.org/examples10.htm) | METHODS  Randomization |
| METHODS  Blinding (masking) | 11 | [Whether or not participants, those administering the interventions, and those assessing the outcomes were blinded to group assignment.](http://www.consort-statement.org/examples11a.htm) When relevant, [how the success of blinding was evaluated](http://www.consort-statement.org/examples11b.htm). | METHODS  Randomization |
| METHODS  Statistical methods | 12 | [Statistical methods used to compare groups for primary outcome(s)](http://www.consort-statement.org/examples12a.htm); [Methods for additional analyses,](http://www.consort-statement.org/examples12b.htm) such as subgroup analyses and adjusted analyses. | METHODS  Statistical methods |
| RESULTS  Participant flow | 13 | [Flow of participants through each stage](http://www.consort-statement.org/examples13a.htm) (a diagram is strongly recommended). Specifically, for each group report the numbers of participants randomly assigned, receiving intended treatment, completing the study protocol, and analyzed for the primary outcome. [Describe protocol deviations from study as planned, together with reasons.](http://www.consort-statement.org/examples13b.htm) | Fig 1  RESULTS  Consort Patient flow |
| RESULTS  Recruitment | 14 | [Dates defining the periods of recruitment and follow-up.](http://www.consort-statement.org/examples14.htm) | Methods – Randomization and Results Patients population |
| RESULTS  Baseline data | 15 | [Baseline demographic and clinical characteristics of each group.](http://www.consort-statement.org/examples15.htm) | Table 1 and  RESULTS  Baseline data |
| RESULTS  Numbers analyzed | 16 | [Number of participants (denominator) in each group included in each analysis and whether the analysis was by "intention-to-treat"](http://www.consort-statement.org/examples16.htm). State the results in absolute numbers when feasible (*e.g*., 10/20, not 50%). | RESULTS  Patient population and Table 2 and 3 |
| RESULTS  Outcomes and estimation | 17 | [For each primary and secondary outcome, a summary of results for each group, and the estimated effect size and its precision](http://www.consort-statement.org/examples17.htm) (*e.g.*, 95% confidence interval). | Fig 2, Table 2,3 and RESULTS  Efficacy SSG vs PM and SSG vs Combination |
| RESULTS  Ancillary analyses | 18 | [Address multiplicity by reporting any other analyses performed](http://www.consort-statement.org/examples18.htm), including subgroup analyses and adjusted analyses, indicating those pre-specified an  RESULTSd those exploratory. | METHODS Statistics and RESULTS  Efficacy SSG vs PM and SSG vs Combination |
| RESULTS  Adverse events | 19 | [All important adverse events or side effects in each intervention group.](http://www.consort-statement.org/examples19.htm) | Table 5 and  RESULTS  Safety |
| DISCUSSION Interpretation | 20 | [Interpretation of the results](http://www.consort-statement.org/examples20.htm), taking into account study hypotheses, sources of potential bias or imprecision and the dangers associated with multiplicity of analyses and outcomes. | DISCUSSION |
| DISCUSSION  Generalizability | 21 | [Generalizability (external validity) of the trial findings.](http://www.consort-statement.org/examples21.htm) | DISCUSSION |
| DISCUSSION  Overall evidence | 22 | [General interpretation of the results in the context of current evidence.](http://www.consort-statement.org/examples22.htm) | DISCUSSION |
